# The Lost Nasopharyngeal Airway: A Sentinel Event to Ponder?

**DOI:** 10.7759/cureus.73606

**Published:** 2024-11-13

**Authors:** Rajashekar R Mudaraddi, Abhay Kuber Bodhey, Naved Ahmed Masood, Hany Fawzi Greiss, Osama Sami Maki Al Ani, Mahmoud Younes Maarouf

**Affiliations:** 1 Anesthesia, Rashid Hospital and Trauma Centre, Mohammed Bin Rashid University of Medicine and Health Sciences, Dubai Health, Dubai, ARE

**Keywords:** computed tomography, endotracheal tube, nasopharyngeal airway, rigid bronchoscope, seizures

## Abstract

Aspiration of the nasopharyngeal airway (NPA) is one of the rarest and life-threatening complications. In the literature, very few cases have been reported. The advent of NPA to protect the airway in semiconscious patients acts like a double-edged sword, based on the patient's condition. The patient factor plays a crucial role. Here, we are reporting a case of aspirated NPA due to the patient's poor gag reflex and human factors at the workplace. The incidence can be minimized if proper handover and documentation by caring personnel are done, which will help reduce the risk of aspiration.

## Introduction

Nasopharyngeal airway (NPA) is one of the common adjuncts used to maintain the patency of the upper airway in patients with compromised airway and semiconscious or conscious patients who are not tolerating oral airway. However, the complications associated with its use are bleeding and aspiration of the NPA in the lower airway [1]. The displacement or aspiration of the NPA into the trachea and bronchi is a very unusual complication [2]. This displacement of devices is more likely to occur in patients with poor airway reflexes secondary to a neurological impairment with a low Glasgow Coma Scale score. It usually presents as sudden respiratory distress with desaturation in a suspected patient [3,4]. Thus, a history and clinical documents may give rise to a clinical suspicion, but the final diagnosis is done through radiological imaging to find the cause of acute respiratory events. The occurrence of this complication can be reduced by selecting properly sized NPA and by securing it in position to avoid dislodgement. This case report also suggests the avoidance of human factors, which can be minimized with proper documentation and handover to the team.

## Case presentation

**Figure 1:**
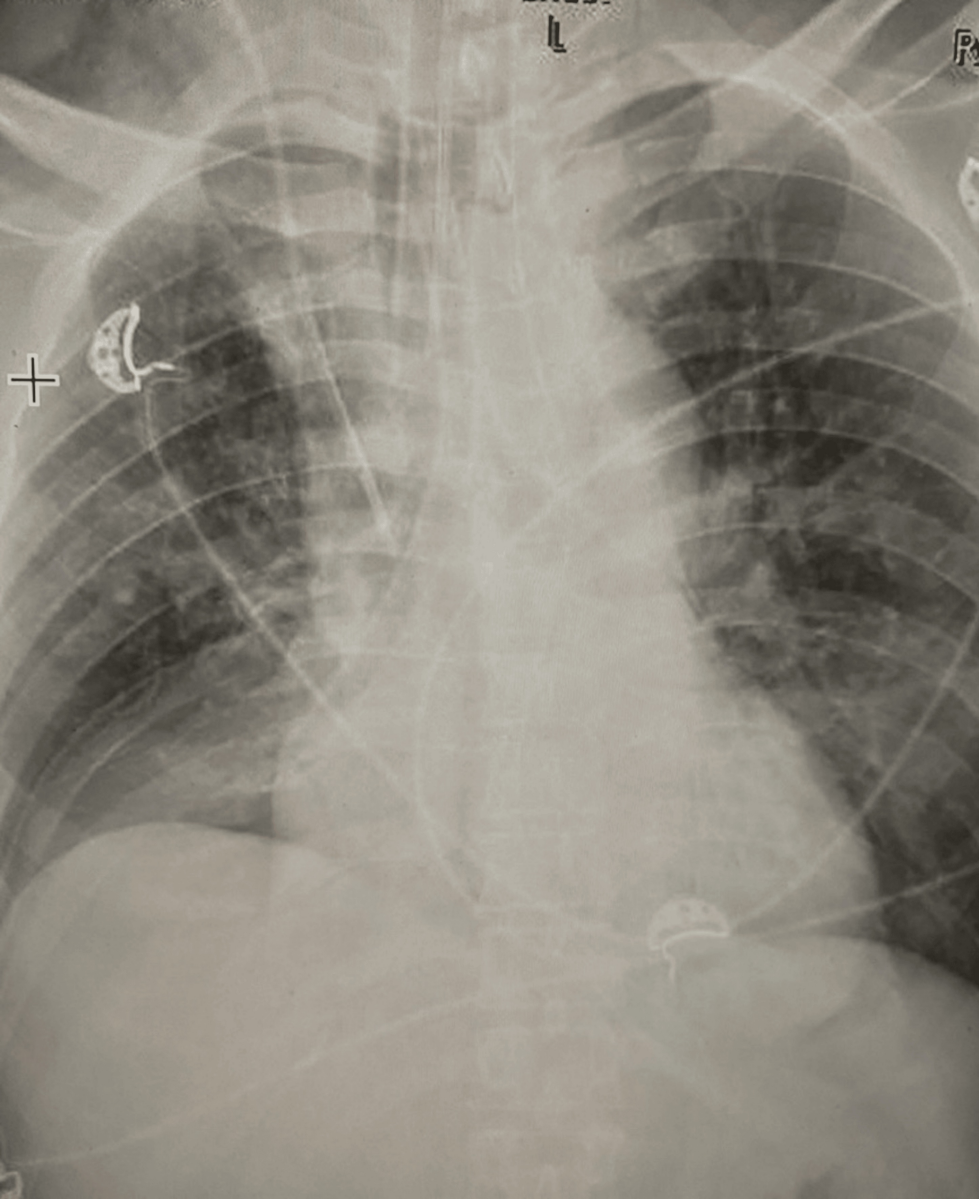
Chest X-ray showing nasopharyngeal airway in the right bronchus.

**Figure 2:**
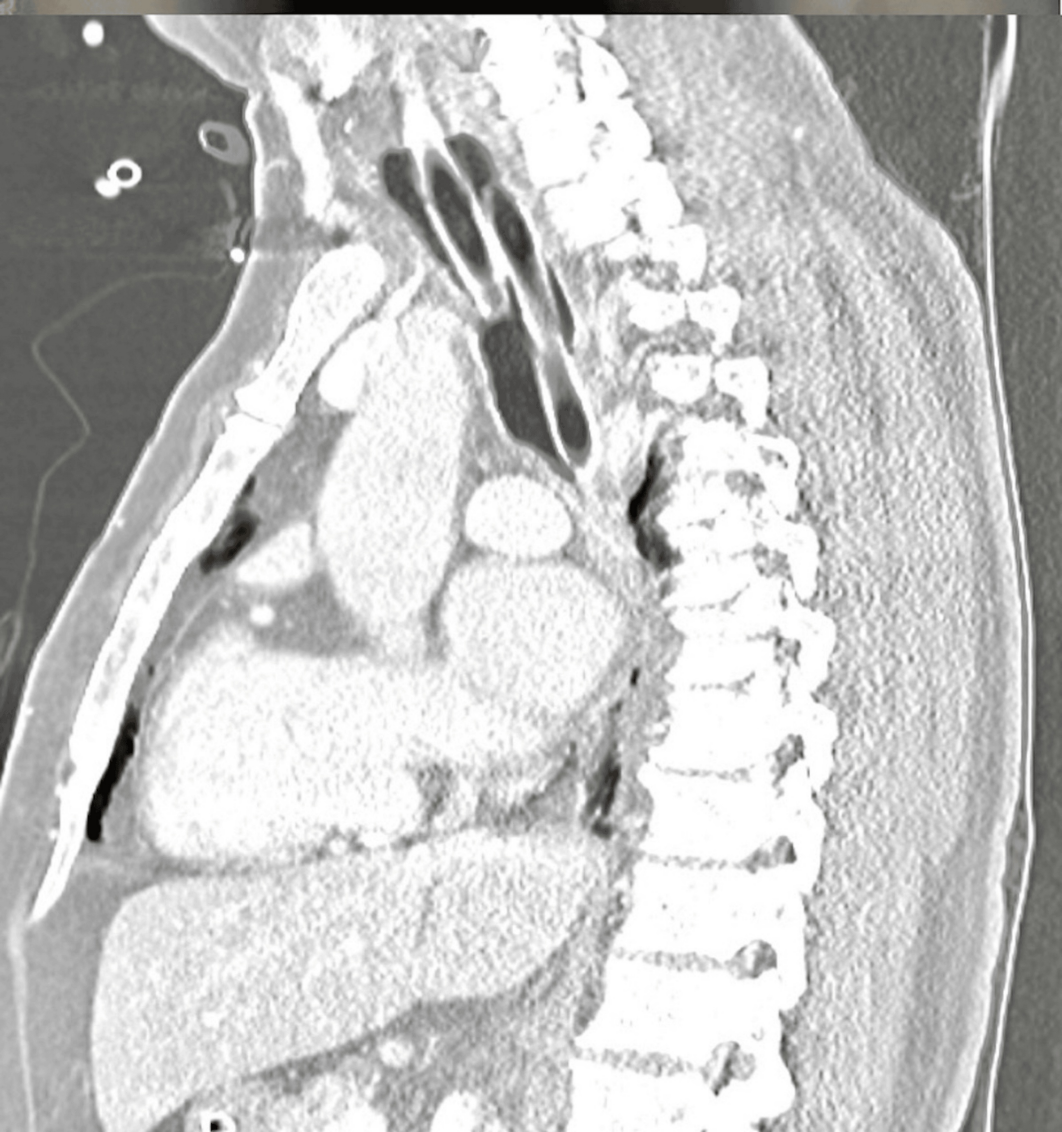
Chest CT showing nasopharyngeal airway in the lateral view.

**Figure 3:**
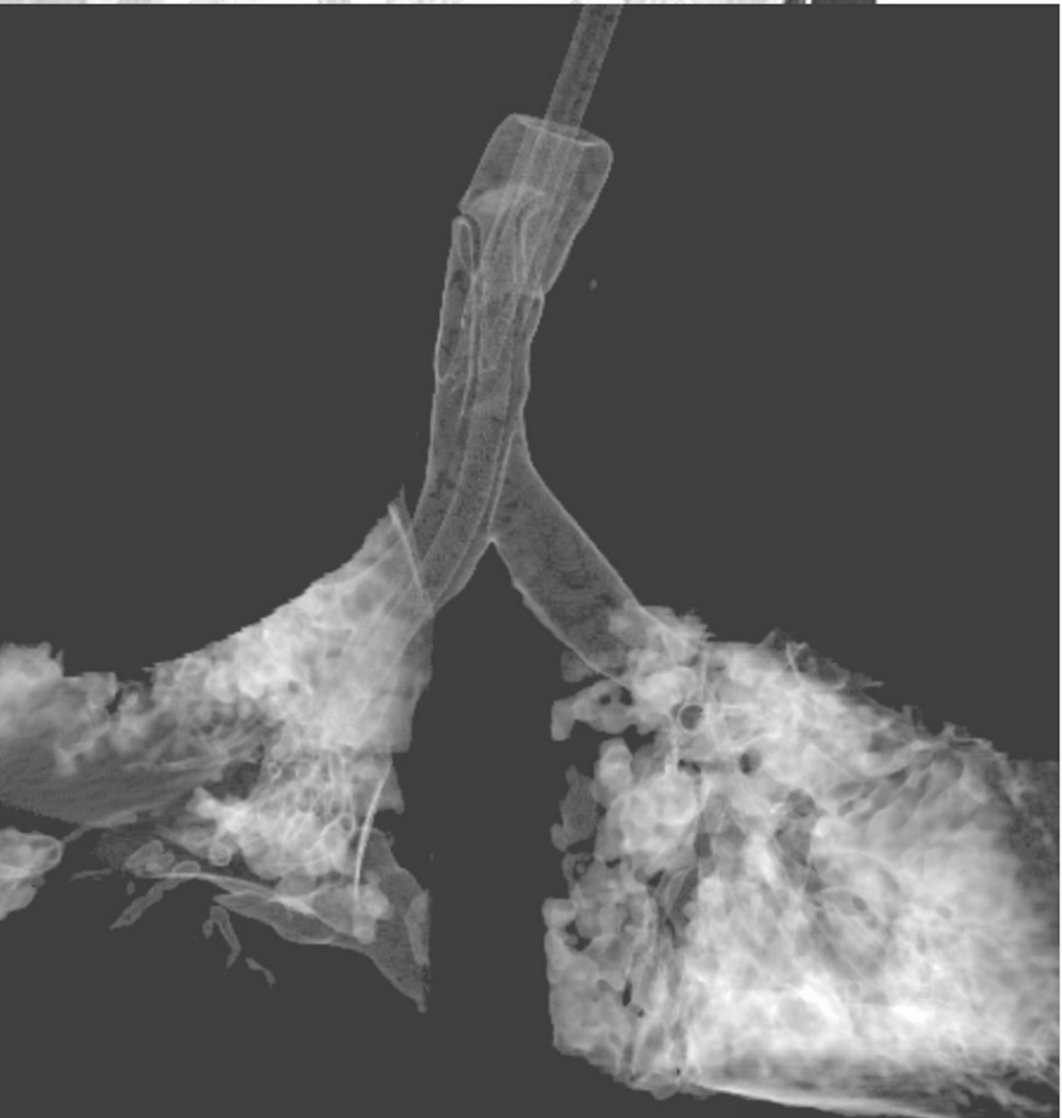
Chest CT reconstructed to show the nasopharyngeal airway.

A 34-year-old male patient presented to the emergency department with seizures, and was initially managed with medications. The patient developed an obstructed airway after intravenous diazepam 10 mg. A No. 7 size NPA was inserted in the left nostril to improve the latency of the airway and breathing. After five hours of insertion, the patient developed sudden desaturation with increased work of breathing. Our team attended a call for intubation. In view of the limited time, our team did emergency intubation and put the patient on a ventilator. As a routine, we did a chest X-ray to confirm the proper positioning of the endotracheal tube (ETT). The chest X-ray (Figure 1) revealed a tube-like structure in the right bronchi apart from the ETT tip above the carina. Then we went through the notes and found the patient had NPA, which was not handed over to the next handover team. The clinical notes were reviewed, and it was realized that the NPA was missing and most likely had dislodged into the bronchus due to the patient's poor consciousness level after the postictal phase with intravenous diazepam. Emergency CT of the chest was done to find out the site of dislodgement of NPA. CT of the chest revealed (Figures 2, 3) the NPA, occupying the whole right bronchi without distal obstruction with both lungs ventilating normally. The decision was made based on CT findings to do an urgent bronchoscopy, in view of the non-availability of a flexible bronchoscope. The patient was shifted to the operating room. The NPA was removed under general anesthesia with a rigid bronchoscope. The patient was shifted to the ICU on a ventilator, eventually extubated, and discharged from the hospital.

## Discussion

NPA aspiration is rare but still can occur. The design of NPA and right bronchial anatomy has led to aspiration into the bronchi instead of the stomach. Several factors contribute to NPA aspiration. First, the NPA design can cause suppleness after lubrication, which leads to dislodgement into the trachea and bronchi. Second, it may occur if the patient may have rubbed his/her nose briefly prior to the incident with poor gag reflex [3-6].

NPA devices are commonly used respiratory adjuncts that are used in clinical practice for a variety of indications. However, they carry the potential risk of becoming aspirated in patients with poor gag reflexes. Complications that may occur in case of NPA aspiration can range from soft tissue injury in the nasal cavity to severe life-threatening airway compromise [7,8]. We highlight the importance of awareness of medical staff regarding the dislodgment and properly securing the NPA device after their placement. The documentation of NPA insertion and proper handover would prevent unanticipated complications and reduce the economic burden on the patient. The device should be removed soon once the cause is treated. Various techniques such as safety pins and tapes can be attached to the flange of the NPA to prevent its distal dislodgement [2]. In the event of aspiration, however, the NPA may be removed using either rigid or flexible bronchoscopy [4]. Rigid bronchoscopy remains a choice for the removal of impacted foreign bodies in the airway. Flexible bronchoscopy is increasingly being used as the first method of intervention in selected cases. A rigid bronchoscope should always be kept as a backup plan when removing a foreign body from the bronchus if a flexible bronchoscope fails to hold the soft part of the NPA. NPA aspiration can be prevented by using a tape at the proximal end of the NPA and securing it to the external nose in the same way as a nasotracheal tube is secured [3-5].

## Conclusions

This case highlights the importance of proper documentation and careful monitoring of NPAs, especially in patients with altered sensorium. We recommend the proper handover, documentation, and training of medical staff to prevent an inadvertent dislodgement of NPAs into the trachea. These measures are essential for patient safety and preventing potential complications associated with airway obstruction. The aspiration of a commonly used NPA is a significant issue for patient safety despite various reasons in the literature. These cases are an indication for re-evaluation of NPA's future design and usage. In the future, NPAs may come with safety features to avoid human and design errors.
